# Autopsy of Joseph Clark, Thirty-Four Hours after Death*The negro, whose upper maxillary bones were removed 16th December. See No. 1, page 7.

**Published:** 1838-04-11

**Authors:** 


### CLINICAL REPORTS.

### PHILADELPHIA HOSPITAL.

*Autopsy of Joseph Clark,* thirty-four hours after death, by* Drs. Gerhard *and* Goddard.

*March 11th,* 1838. Body emaciated; slight infiltration of the extremities, with evident fluctuation of the abdomen; percussion flat, throughout the right side of the chest, except near the clavicle; sonorous, in all the left side anteriorly.

Cranium. The vessels of the dura mater contained rather less than the average quantity of blood; the longitudinal sinus was almost empty, containing no coagulum. Pia mater, remarkably free from blood, both in the large and small vessels; arachnoid membrane, moist, with slight effusion upon its under surface, not sufficient to elevate it from the brain. The membrane on each side of the great fissure, presented a milky opacity, with reddening and increased firmness, in the extent at most, of an inch and a half. A few delicate shreds adhered to the most thickened portion of the membrane, and some old cellular adhesions, without reddening of the membrane, were attached to the arachnoid covering the dura mater, but were easily removed from the convolutions, which were paler than usual. The medullary substance of the cerebrum was almost destitute of vascular traces, remarkably pale, and of average firmness. The ventricles contained about half an ounce of serum. The septum lucidum was slightly softened. Strung along the peduncles of the pineal gland were some hydatids of the size of a millet seed. The thalami and corpora striata were firm; the cortical portion of a darker color than the rest of the brain. The base of the cerebrum was free from adhesion. The arachnoid was smooth, with faint opacity at the anterior portion of the middle lobe of the right hemisphere; there was no softening, or morbid change. The crura cerebri of each side were slightly softened, especially near the margin of the left, which was pulpy, to the depth of a quarter of an inch. Pons Varolii, firm. Cerebellum firm and pale on both sides.

In the middle fossa of the base of the cranium was a tumour, about an inch and three quarters long, and an inch wide. It was covered by the dura mater, which adhered closely to it, but presented no opening. On the side next to the brain, the membrane was smooth, except where it was studded with some small whitish bodies, ten or twenty in number, the largest a quarter of an inch broad, flattened beneath the arachnoid, and adherent to the dura mater. Upon removing the membranes, the tumour was seen to be in contact with the first and third branches of the fifth pair of nerves, while the second branch passed through it.

A vertical and transverse section of the cranium was now made, passing downward on a line with the posterior clinoid process. The lower jaw was disarticulated. The anterior portion of the skull was then removed, and a perpendicular section made through it, passing a little to the right of the septum narium.

*Right Section.* The hard palate was three quarters of an inch in thickness, and was, in a great measure, converted into a scirrhous mass, of the usual cartilaginous consistence. An ulcerated opening existed between the palate process of the superior maxilla and the palate bones. The posterior nares were entirely filled up by the tumour, which penetrated into the sphenoidal sinus, and occupied almost entirely the right half of it. It also passed into the antrum, and extended upwards, as before described, into the middle fossa of the cranium. The turbinated bones were no longer visible. The tumour did not penetrate into the frontal sinus.

*Left Section.* The mucous membrane of the septum narium was injected, and the tumour, on this side, occupied the whole of the nares, penetrating into the antrum. Its consistence was softer than on the right side. It reached as far as the body of the sphenoid bone posteriorly, but did not extend into the sinus. Its extent posteriorly was not so great as on the right side. The lining membrane of the sphenoidal sinus, was of a dark slaty colour, and thickened. The tumour occupied the cellular structure of the ethmoidal bone. Upon subsequent examination, the lymphatic glands of the neck were found to be converted into a semi-cartilaginous mass, nearly resembling true scirrhus.

Thorax. *Right Side.* The pulmonary and costal pleura was of a pale colour, slightly injected near the summit, where it contained in its thickness numerous whitish bodies, of the size of a pin's head, apparently scirrhous. The lung was compressed to one half the usual size, and infiltrated with serum. The quantity of serum, contained in the pleura, was about three pints; it was a little turbid, but with no trace of pus. The adhesions uniting the pleura, were loose and pale; some of them were soft, but the greater part were organized, and contained blood-vessels.

*Left Side.* Pleura nearly free from adhesions, except at the summit, containing a pint of liquid. Lungs hard and firm; adhesions pale, containing bloodless vessels. Tissue of the lungs engorged with serum; containing no tubercular masses, except one or two near the summit, about the size of a pea. A cluster of whitish bodies, apparently cancerous, was situated about the middle of the left lung, near its surface.

The pericardium contained about six ounces of serum.

The left ventricle of the heart was six eighths of an inch thick, rather smaller than usual; the semilunar valves slightly thickened. Aorta pale.

The abdomen contained about two quarts of serum.

The liver was rather less than the usual size; very pale; bile scanty and pale.

Spleen small and firm.

Kidneys firm, pale, free from adhesions.

Stomach pale throughout and softened, containing a liquid resembling a solution of gelatine; pylorus slightly thickened.

The small intestines contained a quantity of thin

* The negro, whose upper maxillary bones were removed 16th December. See No. 1, page 7.

yellowish fœces; membranes not injected, pale. Large intestines also pale.

In the bladder no morbid change; it contained a pint of urine.

Muscles dark and firm.

